# Chandipura virus dysregulates the expression of hsa-miR-21-5p to activate NF-κB in human microglial cells

**DOI:** 10.1186/s12929-021-00748-0

**Published:** 2021-07-07

**Authors:** Neha Pandey, Meghana Rastogi, Sunit K. Singh

**Affiliations:** Molecular Biology Unit, Faculty of Medicine, Institute of Medical Sciences, Banaras Hindu University, 221005 Varanasi, India

**Keywords:** Chandipura virus, Rhabdovirus, MicroRNA, Inflammation, Neuro-inflammation

## Abstract

**Background:**

Chandipura virus (CHPV) is a negative single-stranded RNA virus of the *Rhabdoviridae* family. CHPV infection has been reported in Central and Western India. CHPV causes acute encephalitis with a case fatality rate of 70 % and mostly affects children below 15 years of age. CHPV infection in brain leads to neuronal apoptosis and activation of the microglial cells. The microRNAs (miRNAs) are small endogenous non-coding RNA that regulate the gene expression. Viral infections perturb the expression pattern of cellular miRNAs, which may in turn affect the expression pattern of downstream genes. This study aims to investigate hsa-miR-21-5p mediated regulation of PTEN, AKT, NF-ĸBp65, IL-6, TNF-α, and IL-1β, in human microglial cells during CHPV infection.

**Methods:**

To understand the role of hsa-miR-21-5p in CHPV infection, the human microglial cells were infected with CHPV (MOI-0.1). Real-time PCR, western blotting, Luciferase assay, over-expression and knockdown techniques were used to understand the role of hsa-miR-21-5p in the regulation of PTEN, AKT and, NF-ĸBp65, IL-6, TNF-α, and IL-1β in this study.

**Results:**

The hsa-miR-21-5p was found to be upregulated during CHPV infection in human microglial cells. This led to the downregulation of PTEN which promoted the phosphorylation of AKT and NF-ĸBp65. Over-expression of hsa-miR-21-5p led to the decreased expression of PTEN and promoted further phosphorylation of AKT and NF-ĸBp65 in human microglial cells. However, the inhibition of hsa-miR-21-5p using hsa-miR-21-5p inhibitor restored the expression.

**Conclusions:**

This study supports the role of hsa-miR-21-5p in the regulation of pro-inflammatory genes in CHPV infected human microglial cells.

**Supplementary Information:**

The online version contains supplementary material available at 10.1186/s12929-021-00748-0.

## Background

Chandipura Virus (CHPV) was first identified in 1966 by National Institute of Virology, Pune, in Chandipura village, Maharashtra, India [1]. CHPV is a negative single-stranded RNA virus of the *Rhabdoviridae* family [2]. CHPV infection is one of the most unattended and less-studied class of viral infections from molecular pathogenesis point of view. CHPV is transmitted through the sandflies *Phlebotomus* sp. or *Sergentomyia sp* and the mosquito *Aedes aegypti* [3]. The clinical manifestations of CHPV infection start as an influenza-like illness, associated with abdominal pain, vomiting, altered consciousness and impaired neurological functions [4]. CHPV invades the nervous system and causes acute encephalitis with a high fatality rate of 55-70 % [4, 5]. The patients sera and cerebrospinal fluid (CSF) have been used to detect CHPV through RT-PCR [6]. Neurons are the primary sites for CHPV replication [7]. Progressive replication has been reported in the brain and spinal cord. CHPV infected neurons undergo Fas-ligand mediated apoptosis through the extrinsic apoptotic pathway [5, 7]. CHPV infection also affects the cholesterol homeostasis in the brain for its maturation and release [7–9]. The microglia are the resident macrophages of the brain that quickly respond to various trauma, injuries, and infections of the Central Nervous System (CNS) [10]. CHPV infection leads to the activation of the microglial cells, which further results to the increased production of inducible nitric oxide synthase (iNOS), Reactive oxygen species (ROS), Cyclooxygenase − 2, Nitric Oxide (NO) in the mouse brain [11, 12]. CHPV infection has been reported to activate the MAPK p38, JNK1&2 NF-ĸB pathway and the increased expression of pro-inflammatory cytokines and chemokines in microglia [13]. A nitrosporeusine analogue has been reported to suppress the NF-ĸB activation during CHPV infection in microglial cells [13]. A recent study indicated that CHPV required NF-ĸB subunit Rel-A for its replication [14]. The study showed that the lack of Rel A/NF-ĸB p65 suppresses CHPV replication [14].

The microRNAs are small non-coding RNAs that regulate the gene expression by targeting the 3’UTR of mRNAs [15]. The miRNAs have been reported for their roles in the gene regulation activity during direct viral infections and/ or through bystander mechanism [16]. It has been reported that CHIKV exploits hsa-miR-146a to modulate the host’s immune response [17]. Similarly, miR-375 has been reported to regulate REL-1 in Dengue virus replication [18]. miR-432 has also been reported to modulate the inflammatory responses during Japanese Encephalitis virus (JEV) infection [19]. The PI3K/AKT pathway acts as a central regulator of various cellular processes [20]. Dysregulation of PI3K/AKT signalling has been reported in several metabolic disorders, cancers, and inflammation [20, 21]. The PI3K/AKT signalling has been reported to be a crucial component in LPS mediated activation of microglial cells [22].The PI3K/AKT signalling has been involved in regulating viral replication, viral entry, host’s response to viral infection and virus-induced apoptosis [23–27]. The dysregulation of PI3K/AKT signalling has been reported in several viral infections. The Phosphatase and tensin homolog (PTEN) is a lipid and protein phosphatase that negatively regulates the activation of PI3K/AKT signalling [28]. PTEN has been reported to play roles in regulating immune responses and antiviral immunity [29, 30]. The PTEN mediated regulation of the PI3K/AKT/IRF-3 axis has also been reported in JEV infected microglial cells [27].We previously reported the miRNA expression profile (miRnome) of CHPV infected human microglial cells [31]. Recent studies have highlighted the role of hsa-miR-21-5p in the regulation of inflammatory responses in macrophages [32]. In this study, we demonstrated the role of hsa-miR-21-5p in activation of inflammatory responses in CHPV infected microglia. The suppression of PTEN by hsa-miR-21-5p activated the PI3K/AKT signalling and promoted the activation of NF-ĸB and pro-inflammatory responses during CHPV infection.

## Materials and methods

### Cell culture

The human microglial cells (CHME3) were received as a gift from Prof. Anirban Basu from National Brain Research Centre (NBRC), Manesar, India. The CHME3 cells and Vero cells were cultured in Dulbecco’s Modified Eagle’s Medium (DMEM) (#12100-038 Gibco) supplemented with 10 % fetal bovine serum (#10270106 Gibco) and 1000U/ml of PENSTREP- L-glutamine (#10378 Gibco). Both the cells were maintained at 37 °C in a humidified CO_2_ incubator having a constant supply of 5 % CO_2_.

### Virus propagation, titration, and infection

The CHPV strain 1653514, and JEV strain JaOArS982 obtained from NBRC Manesar, India was propagated *in-vitro* in Vero cells and PS cells respectively. Chikungunya virus (CHIKV) Ross Strain E1: A226 was received as a kind gift from Professor Duane Gubler (Emerging Infectious Disease Program, Duke-NUS Medical School, Singapore). Vero cells were infected by CHPV at a MOI of 0.01 in incomplete DMEM. The incomplete DMEM was replaced by complete DMEM three hours post-infection. The cells were placed in a 5 % CO_2_ incubator for two days or until 70 % cell death was observed. For propagation of CHIKV, Vero cells were infected with CHIKV and supernatant was collected 72 h post-infection. The cell culture supernatant was collected at 5000 X g for 15 min to remove the cell debris and stored at -80 °C till further use. Viral plaque assay was done to determine the viral titer of *in-vitro* propagated CHPV and CHIKV. The supernatant was diluted to 10 times in incomplete DMEM. The dilutions were added to the monolayer of Vero cells grown on a 6-well plate. The supernatant was removed from the cells and cells were washed with 1X PBS twice, 2 h post-infection. The cells were then over-layered with 2ml of 2 % Low Melting Agarose (LMA) in 2X DMEM supplemented with 10 % FBS. The cells were kept at 5 % CO_2_, 37 °C 16 h for CHPV and 72 h for CHIKV, after which the cells were fixed with 10 % paraformaldehyde at room temperature for 4 h, followed by removal of the agarose overlay. The plaques were stained with 0.1 % crystal violet stain, and the virus titer was determined as the plaque-forming units (PFU) per millilitre of the supernatant as described elsewhere [19].

The human microglial cells were plated in a 6 well plate at a cell density of 0.5 × 10^6^ cells/ well. After attaining the uniform monolayer, the human microglial cells were infected with CHPV at a MOI of 0.1 in incomplete DMEM for two hours at 37 °C in 5 % CO_2_ incubator [8, 12, 13, 31]. The cells were washed with 1X PBS and supplemented with complete DMEM after two hours of infection. Then the cells were harvested 24 h post-infection and stored at -80 °C till further use [31].For CHIKV infection human microglial cells were plated in a 6 well plate at a cell density of 0.3 × 10^6^ cells/ well. The microglial cells were infected with CHIKV at MOI of 2 in incomplete DMEM for two hours at 37 °C in 5 % CO_2_ incubator. The cells were washed with 1X PBS and supplemented with complete DMEM after two hours of infection. The cells were then harvested at 24-, 32- and 48-hours post-infection and stored at -80 °C till further use. JEV infection was given in microglial cells as described previously [33].

### Total RNA isolation and quantitative Reverse transcription PCR

Total RNA was isolated using the miRNeasy kit (#217004 Qiagen; Netherlands). complementary DNA (cDNA) was synthesized from the isolated RNA using the Superscript reverse transcriptase system (#11904-018, Invitrogen, CS, USA) using the manufacturer’s protocol. Thermal cycle for reverse transcription was as follows: 65 °C-5 min, 25 °C-10 min, 42 °C-50 min, and 70 °C-10 min, followed by RNase H treatment for 20 min at 37 °C for removal of residual RNA. The confirmation of CHPV infection in human microglial cells was done by qPCR (Agilent AriaMx) with primers specific for viral Phosphoprotein gene and was normalized to gene expression levels of GAPDH. The gene expression levels of TNF-alpha, IL-1β, and IL-6 were also observed through qPCR by using Agilent Brilliant III ultrafast SYBR green master mix (#600882, Agilent Technologies, California, US). The sequences of Primers used have been described in Table 1.

**Table 1:** List of Primers, miRNA oligos and scramble sequence

**CHPV P Protein Forward**	**5’ACCTGGCTCCAAATCCAATAC3’**
CHPV P Protein Reverse	5’ GGTGGATCAGACGGAGAGATA 3’
GAPDH Forward	5′ ATGGGGGAAGGTGAAGGTCG 3′
GAPDH Reverse	5′ GGGGTCATTGATGGCAACAATA 3′
IL-6 Forward	5′ ACTCACCTCTTCAGAACGAATTG 3′
IL-6 Reverse	5′ CCATCTTTGGAAGGTTCAGGTTG 3’
TNF-α Forward	5′ CCTCTCTAATCAGCCCTCTG 3′
TNF-α Reverse	5′ GAGGACCTGGGAGTAGATGAG 3′
IL-1β Forward	5’AACCTGCTGGTGTGTGACGTTC3’
IL-1β Reverse	5’CAGCACGAGGCTTTTTTGTTGT3’
Mimics of hsa-miR-21-5p	5’ UAGCUUAUCAGACUGAUGUUGA3’
Scramble of hsa-miR-21-5p	5’CAAUAAAAUACUACCAUGUUGA3’

### The miRNA expression in CHPV infected human microglial cells

The hsa-miR-21-5p expression was studied by using the TaqMan microRNA assay kit which included the 5x primer and 20x TaqMan probe for the detection (Assay ID# 000397, ABI). The cDNA was prepared by using MultiScribe TaqMan Reverse Transcriptase (#4366596; Applied Biosystems) and 5x primer following the manufacturer’s protocol. The 20x TaqMan probe and the TaqMan Universal PCR master mix (#4324018; Applied Biosystems) was used to detect the microRNA expression levels. The miRNA expression was normalized to the expression levels of RNU6b.

### The microRNA overexpression

The miRBase was used to obtain the sequence of mature hsa-miR-21-5p. This sequence, along with a scramble sequence of hsa-miR-21-5p was commercially synthesized by Imperial Life Sciences. The sequence of RNA oligos has been provided in Table 1. The CHME3 cells were seeded in a 6 well plate at a cell density of 0.4 × 10^6^ cells/ well a day before transfection. Transfection of CHME3 cells was done in commercial low serum media Opti-MEM (#31985070 Gibco) using Lipofectamine2000 (#11668-019; Invitrogen) with 100 picomoles of hsa-miR-21-5p mimic and 100 picomoles of hsa-miR-21-5p scramble. The sequence of hsa-miR-21-5p mimic and scramble have been provided in Table 1. The Opti-MEM was replaced by complete DMEM 6 h post-transfection. The CHPV infection was given 24 h post-transfection. The cells were harvested 48 h post-transfection. The overexpression of hsa-miR-21-5p was confirmed by qPCR using the TaqMan miRNA assay.

### The microRNA inhibition studies

The hsa-miR-21-5p inhibitor (#MH10206; Invitrogen) was used to inhibit the expression of hsa-miR-21-5p in this study. The CHME3 cells, seeded as described previously in the miRNA overexpression experiment, were transfected with 100 picomoles of the miRNA inhibitor and Cy3 labelled control anti-miR using Lipofectamine2000 (#11668-019; Invitrogen). The transfection was carried out in commercial low serum media Opti-MEM (#31985070; Gibco) which was later replaced with complete DMEM 6 h post-transfection. The CHPV infection was given 24 h post-transfection and cells were harvested after the completion of 48 h. The inhibition of hsa-miR-21-5p was confirmed by qPCR using the TaqMan miRNA assay.

### Western blotting

The cell pellets harvested during infection and transfection experiments were lysed using RIPA buffer (#786 − 489; G Biosciences) and Protease/Phosphatase Inhibitor Cocktail (#5872, CST). The total protein was quantified using the Bicinchoninic Acid *(*BCA*)* Protein Assay (#786 − 570, G-Biosciences). The protein samples were loaded equally on each well and were resolved on 10 % SDS gel. The resolved proteins were later transferred on PVDF membrane (*#* IPFL00010, Merck) at 100 V for 2-2.5 h. The membranes were blocked in 5 % BSA (#MB083; Himedia) for an hour and later incubated in Primary antibody diluted in 5 % BSA overnight at 4 °C. The membranes were washed thrice with 1X TBST for 15 min each and later incubated in HRP conjugated secondary antibodies for two hours at room temperature. The following antibodies and dilutions were used for Western Blotting: anti-PTEN antibody (# 9559 S CST) dilution 1:2000, the anti-β-tubulin antibody (#2146S; CST) dilution 1:3000, anti-AKT antibody (#2920S; CST) dilution 1:2000, anti-p-AKT antibody (#9271; CST) dilution 1:2000, anti-p-NF-κB p65 (ser275) antibody (#PA5-37718; Invitrogen) dilution 1:2000 and anti- NF-κB p65 antibody (#4764S; CST) dilution 1:1000, anti-IL-6 (# ITT06087; G-Biosciences) dilution 1:1000, anti TNF-α (# ITT07014; G-Biosciences) dilution 1:1000. The treatment of goat anti-rabbit and rabbit anti-mouse secondary antibodies were given at dilution of 1: 40,000. The membranes were washed thrice with 1X TBST and were developed in ChemiDoc (Azure Biosystems) using the femtoLUCENT PLUS-HRP (#786-003; G-Biosciences) at different exposures. The experiments were done in triplicates and Image J Software (1.42 vs.) was used to analyse the images.

### Luciferase assay

The effect of hsa-miR-21-5-p on the promoter activity of NF-κB was studied through luciferase assay. The NF-κB plasmid was used at a concentration of 1 µg/mL and β-gal at a concentration of 700ng/mL. The plasmids were co-transfected in CHME3 cells with 100 picomoles of hsa-miR-21-5-p mimic, scramble sequence (scr), inhibitor and Cy3 for 48 h. The cells were infected with CHPV after 24 h of transfection at MOI 0.1. The luciferase expression was studied by using the Luciferase assay kit (#E4030; Promega) as per the manufacturer’s protocol and the luminescence activity was measured on the multimode reader (Synergy HTX, Bio-Tek). β-gal plasmid vector was used to normalize the luminescence values. The β-galactosidase activity was measured by a plate reader (imark plate reader, Bio-Rad) at the absorbance of 420nm.

### Statistical analysis

The data have been expressed as the mean ± SE from three independent biological experiments. The one-way ANOVA and student t-test have been used to calculate the p-values (< 0.05 was considered significant), where *p < 0.05, **p < 0.01, and ***p < 0.001 by using GraphPad Prism (ver 8.3.0). More than two independent variables were compared using one-way ANOVA, while the student t-test was used for comparing only two-variables: (control vs. test).

## Results

### The regulation of PI3K/AKT pathway through hsa-miR-21-5p in CHPV infected human microglial cells

**Fig. 1:**
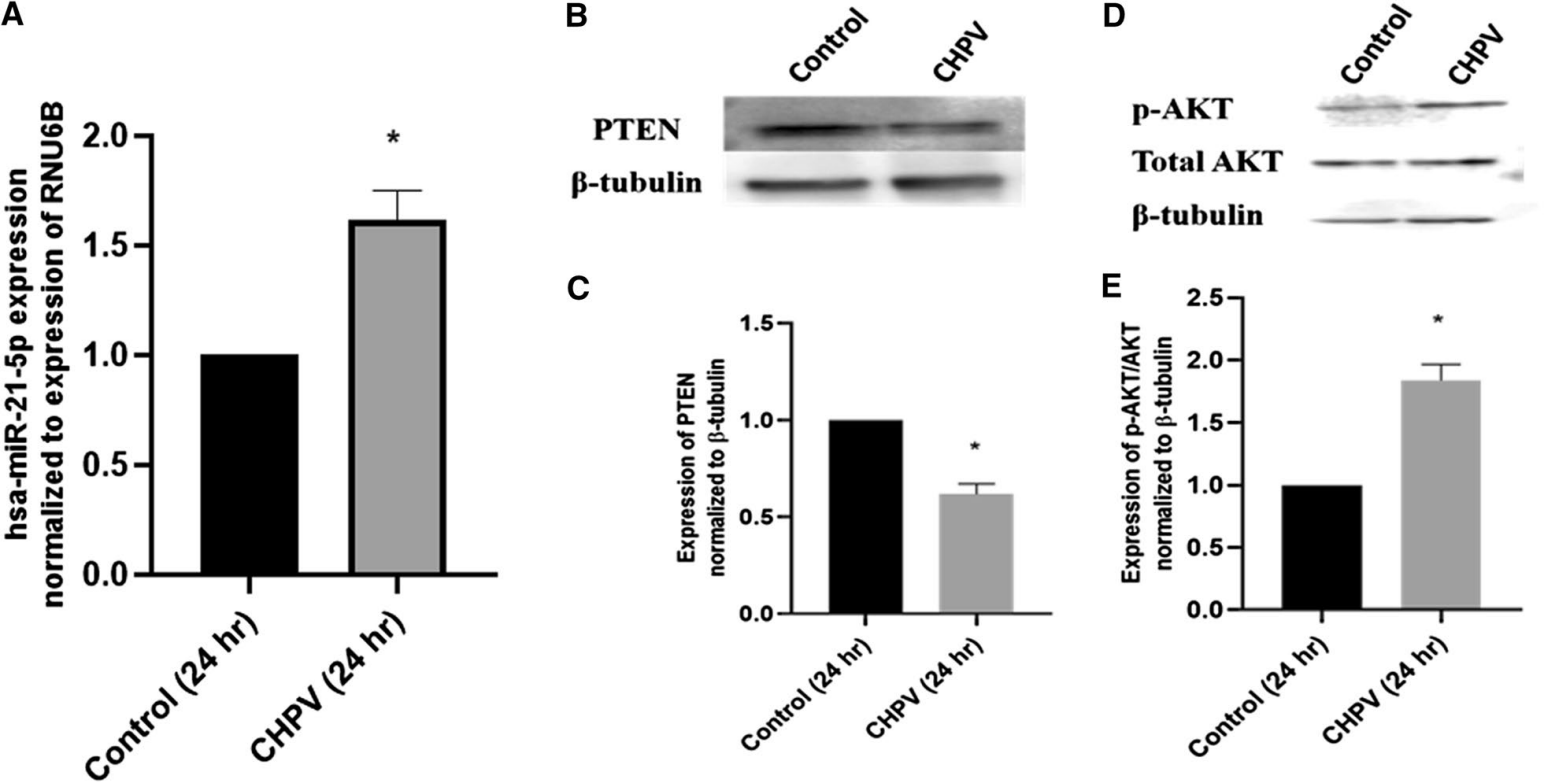
The regulation of PI3K/AKT pathway through hsa-miR-21-5p in CHPV infected human microglial cells. The human microglial cells were infected with CHPV at MOI 0.1 and were harvested 24 h post infection. **A** The expression of hsa-miR-21-5p was checked by qPCR using TaqMan primers and probes specific for miR-21-5p. The expression of hsa-miR-21-5p was normalized to the expression of RNU6B and was 1.6 folds upregulated compared to Control. **B** Western Blot analysis showed downregulation of PTEN expression in human microglial cells. **C** The graph displays the densitometry analysis of PTEN normalized to β-tubulin and showed significant decrease of 40 % in PTEN expression. **D** Western Blot analysis showed induction in p-AKT/AKT expression. **E** The graph of densitometry analysis of p-AKT/AKT showed significant increase of 1.8 folds in p-AKT expression. The experiments were performed in triplicate (n = 3) and shown as SE ± mean. Student unpaired t test has been applied by using GraphPad-prism 8 (ver 8.3.0) to calculate the *p*-values, where: *p < 0.05, **p < 0.01, and ***p < 0.001

Cellular microRNAs are known to regulate the cellular immune responses during viral infection [16]. The hsa-miR-21-5p has previously been reported to regulate the inflammatory responses in macrophages [32]. We investigated the role of increased expression of hsa-miR-21-5p during CHPV infection. CHPV infection was given to human microglial cells at MOI 0.1 for 12 h, 18 h and 24 h. (Additional file 1: Figure S1A, C, D, Additional file 2: Figure S2). CHPV infection significantly increased the hsa-miR-21-5p expression to 1.6 folds at 24 h in human microglial cells (Fig. 1A).

Many studies previously reported the involvement of the PI3K/AKT pathway in the host immune responses during viral infections and cancers [32]. To understand the effect of hsa-miR-21-5p on the PI3K/AKT pathway during CHPV infection, we checked the abundance of PTEN, a previously reported target of hsa-miR-21-5p and negative regulator of PI3K /AKT signalling. (Additional file 1: Figure S1B, E, F) This targeting was confirmed using hsa-miR-21-5p mimic and inhibitor. PTEN expression was 39 % downregulated during CHPV infection (Fig. 1B, C). In addition, CHPV infection promoted the phosphorylation of AKT. The protein levels of pAKT/AKT were increased by 1.8 folds during CHPV infection (Fig. 1D, E).

### CHPV infection activates NF-κB in human microglial cells

**Fig. 2:**
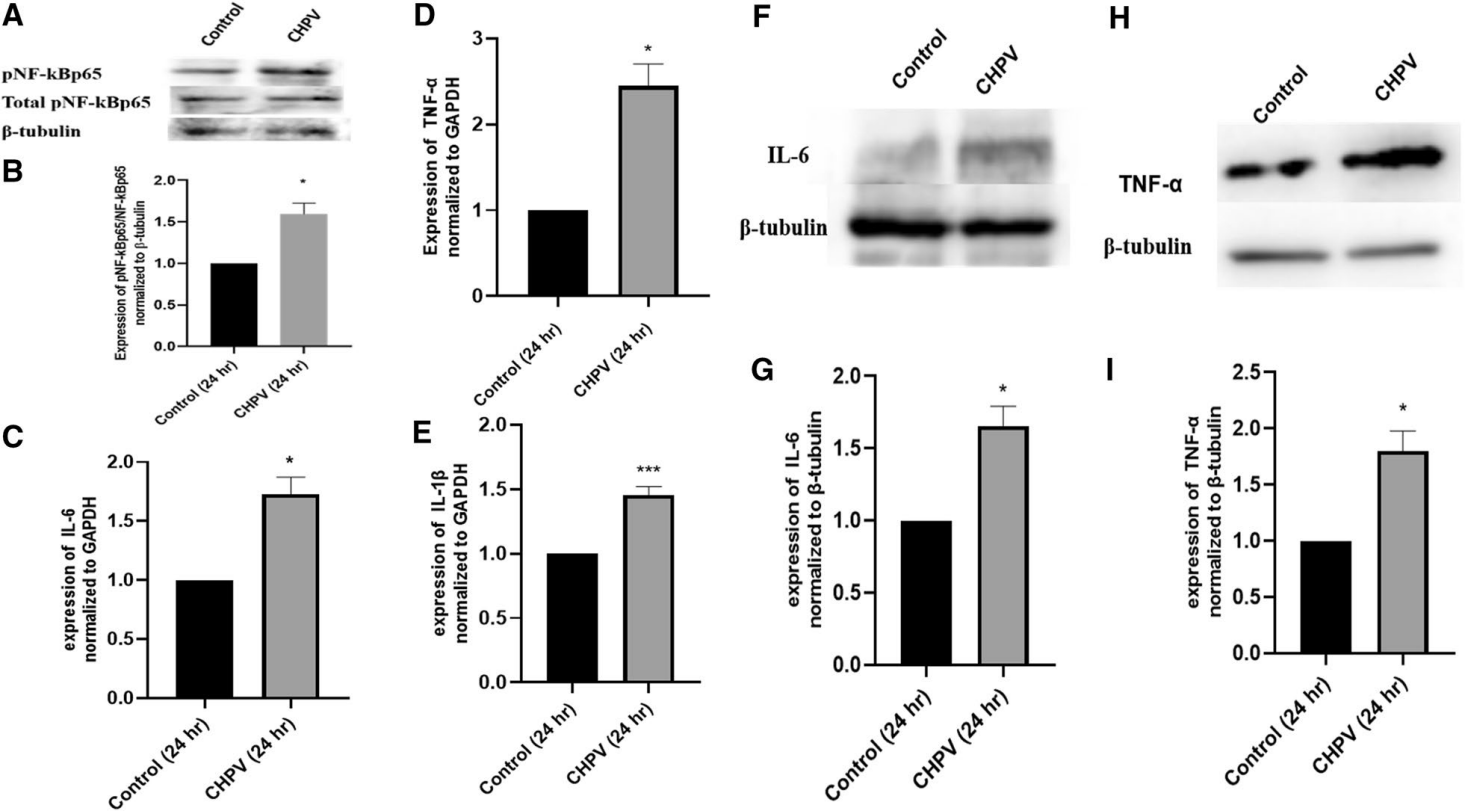
CHPV infection activates NF-κB in human microglial cells. CHPV infection promotes phosphorylation of NF-κBp65 which translocate to nucleus and induces the expression of IL-6 and TNF-α. **A** Western Blot analysis showed increased expression of pNF-κBp65 in human microglial cells. **B** The densitometry analysis showed that the expression of pNF-κBp65 increased to 1.5 folds following CHPV infection in microglia. **C** The expression of IL-6 24 h post CHPV infection, was checked using qPCR. The expression of IL-6 was normalized to the expression of GAPDH and was 1.6 folds upregulated compared to Control **D** The expression of TNF-α 24 h post CHPV infection, was checked using qPCR. The expression of TNF-α was normalized to the expression of GAPDH and was 2.4 folds upregulated compared to Control. **E** The expression of IL-1β 24 h post CHPV infection, was checked using qPCR. The expression of IL-1β was normalized to the expression of GAPDH and was 1.45 folds upregulated compared to Control. **F** Western Blot analysis during CHPV infection showed increased expression of IL-6 in human microglial cells. **G** The densitometry analysis showed that the expression of IL-6 increased following CHPV infection in microglia. **H** Western Blot analysis showed increased expression of TNF-α in human microglial cells. **I** the densitometry analysis showed that the expression of TNF-α increased following CHPV infection in microglia. The experiments were performed in triplicate (n = 3) and shown as SE ± mean. Student unpaired *t* test has been applied using by using GraphPad-prism 8 (ver 8.3.0) to calculate the *p*-values, where: *p < 0.05, **p < 0.01, and ***p < 0.001

NF-kB is a transcription factor that regulates the expression of genes related to inflammation [34]. The hsa-miR-21-5p has been previously reported to enhance the NF-kB by downregulating the PTEN in certain cancers. CHPV infection has also been reported to promote the NF-kB activation during the early hours of infection [13]. In this study, we observed the NF-kB activation at 24 h. The protein levels of pNF-kBp65/NF-kBp65 were found to be increased by ~ 1.5 folds during CHPV infection in microglia (Fig. 2A, B). In addition, the gene expression at mRNA levels of IL-6, TNF-α and IL-1β were found to be increased by 1.7 folds, 2.4 and 1.45 folds respectively during CHPV infection. (Fig. 2C–E). In addition, we found the increased expression of IL-6 and TNF-α at protein level too, by western blotting in CHPV infected human microglial cells at 24 h (Fig. 2F–I).

### The effect of hsa-miR-21-5p overexpression in microglial cells during CHPV infection

**Fig. 3:**
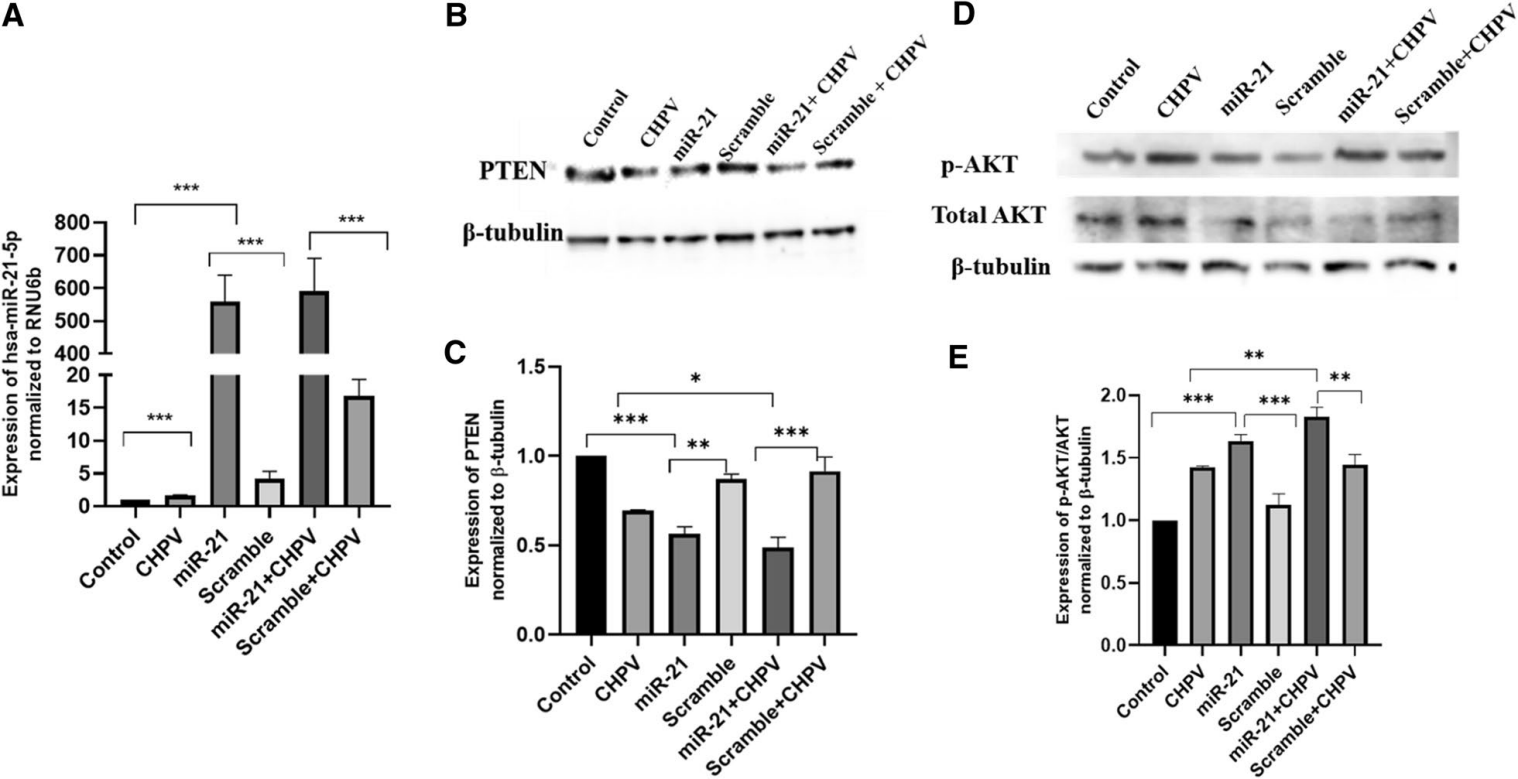
The effect of hsa-miR-21-5p overexpression in microglial cells during CHPV infection. The hsa-miR-21-5p was over-expressed in human microglial cells followed by CHPV infection.** A **The over-expression of miR-21 was confirmed by qPCR using TaqMan primers and probes specific for hsa-miR-21-5p. The expression of hsa-miR-21-5p was normalized to the expression of RNU6B. The hsa-miR-21-5p increased by 590 folds in hsa-miR-21-5p + CHPV compared to CHPV, while hsa-miR-21-5p was 550 folds upregulated compared to scramble.** B **Western Blot analysis showed downregulation of PTEN expression after over-expression of hsa-miR-21-5p.** C **The graph displays the densitometry analysis of PTEN normalized to β-tubulin and showed significant decrease of 20 % in hsa-miR-21-5p + CHPV compared to CHPV, while hsa-miR-21-5p was 30 % downregulated compared to scramble. **D **Western Blot analysis showed induction in p-AKT/AKT expression post over-expression of miR-21 and infection in human microglial cells.** E **The graph of densitometry analysis of p-AKT/AKT showed significant increase of 30 % in p-AKT expression in hsa-miR-21-5p + CHPV compared to CHPV, while hsa-miR-21-5p was 30 % upregulated compared to scramble. The experiments were performed in triplicate (n = 3) and shown as SE ± mean. One-way ANOVA has been applied by using GraphPad-prism 8 (ver 8.3.0) to calculate the p-values, where: *p < 0.05, **p < 0.01, and ***p < 0.001

The hsa-miR-21-5p was over-expressed during CHPV infection in microglial cells by using hsa-miR-21-5p mimics and scrambled sequence of hsa-miR-21-5p as a positive control (Fig. 3A). The expression of PTEN suppressed by ~ 30 % in hsa-miR-21-5p mimic transfected microglial cells compared to cells transfected by scrambled sequence. The overexpression of hsa-miR-21-5p along with CHPV infection decreased the expression of PTEN by 20 % compared to CHPV infection (Fig. 3B, C). The expression of pAKT/AKT increased in hsa-miR-21-5p over-expression by ~ 30 % as compared to scramble. In hsa-miR-21-5p over-expression + CHPV, p-AKT/AKT expression was ~ 30 % higher compared to CHPV (Fig. 3D, E).

### The effect of hsa-miR-21-5p knockdown in microglial cells during CHPV infection

**Fig. 4:**
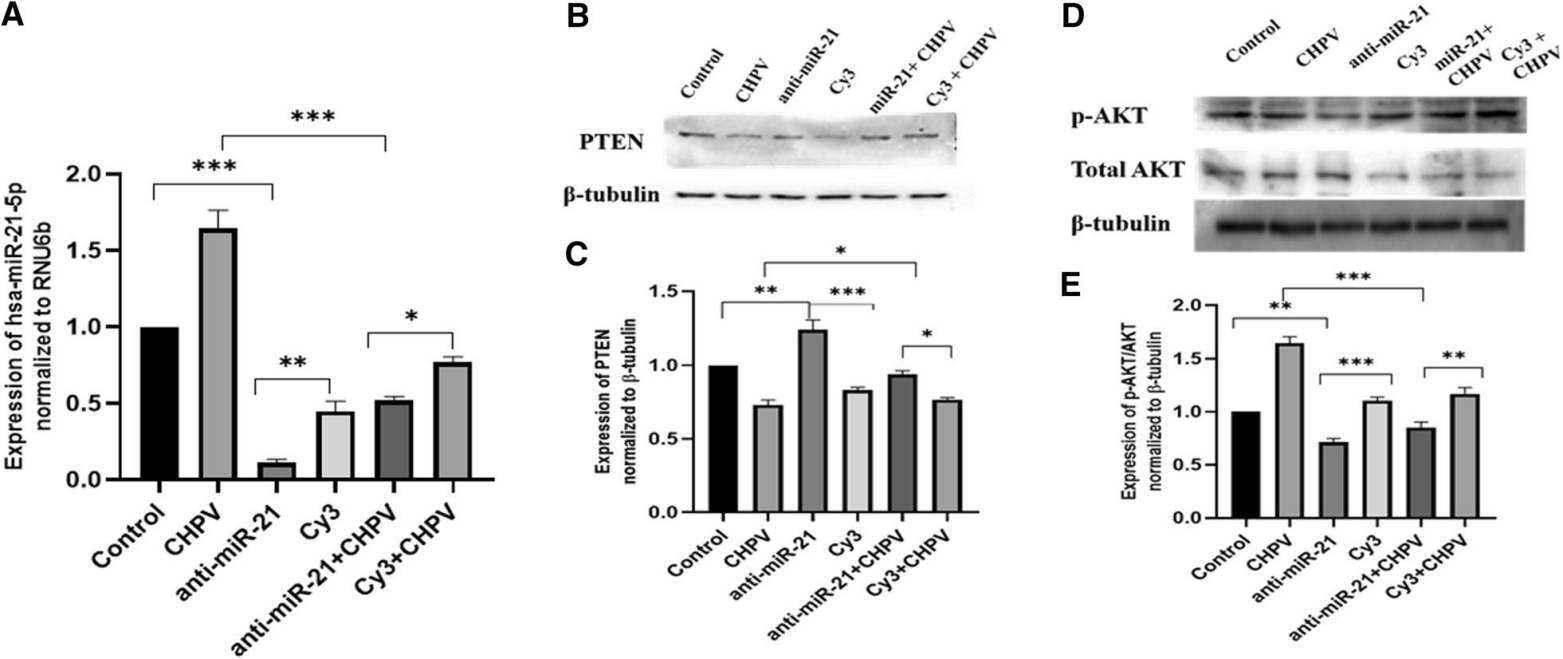
The effect of hsa-miR-21-5p knockdown in microglial cells during CHPV infection. The hsa-miR-21-5p was knockdown and CHPV infection was given in human microglial cells.** A **The knockdown of hsa-miR-21-5p was confirmed by qPCR using TaqMan primers and probes specific for hsa-miR-21-5p. The expression of anti-miR-21-5p was normalized to the expression of RNU6B. The hsa-miR-21-5p decreased by 70 % in anti-miR-21-5p + CHPV compared to CHPV, while anti-miR-21-5p itself was 30 % downregulated compared to Cy3. **B **Western Blot analysis showed that PTEN expression was restored following knockdown of hsa-miR-21-5p. **C **The graph displays the densitometry analysis of PTEN normalized to β-tubulin and showed significant recovery of 20 % in anti-miR-21-5p + CHPV compared to CHPV, while anti-miR-21-5p alone increased the PTEN expression by 30 % compared to Cy3. **D **Western Blot analysis showed restoration of p-AKT/AKT expression post knockdown of hsa-miR-21-5p and CHPV infection in human microglial cells. **E **The graph of densitometry analysis of p-AKT/AKT showed significant decrease of 50 % in p-AKT expression in anti-miR-21-5p + CHPV compared to CHPV, while p-AKT/AKT expression in anti-miR-21-5p alone was 28 % downregulated compared to Cy3. The experiments were performed in triplicate (n = 3) and shown as SE ± mean. One-way ANOVA has been applied using by using GraphPad-prism 8 (ver 8.3.0) to calculate the *p*-values, where: *p < 0.05, **p < 0.01, and ***p < 0.001

The hsa-miR-21-5p was knocked down during CHPV infection using anti-hsa-miR-21-5p (Fig. 4A). The PTEN expression was ~ 30 % higher in anti-hsa-miR-21-5p as compared to Cy3. PTEN expression was restored by ~ 20 % in anti-hsa-miR-21-5p + CHPV compared to CHPV (Fig. 4B, C). The pAKT/AKT expression got suppressed by ~ 28 % in anti-hsa-miR-21-5p compared to Cy3. In presence of CHPV infection, the p-AKT/AKT expression got restored by ~ 50 % in anti-hsa-miR-21-5p + CHPV and CHPV (Fig. 4D, E).

### CHPV infection promotes pro-inflammatory responses in human microglial cells

**Fig. 5:**
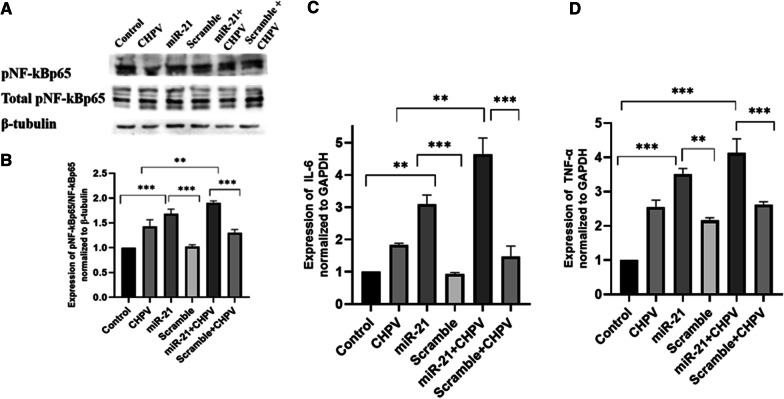
The effect of hsa-miR-21-5p over-expression on NF-κB activation. The effect of hsa-miR-21-5p on NF-κB activation was studied during over-expression and CHPV infection in human microglial cells. **A **Western Blot analysis showed induction in pNF-κB/NF-κB expression post over-expression of hsa-miR-21-5p and infection in human microglial cells.** B **The graph of densitometry analysis of pNF-κB/NF-κB showed significant increase of pNF-κB expression by 45 % in hsa-miR-21-5p + CHPV compared to CHPV, while hsa-miR-21-5p alone was upregulated 60 % compared to scramble. **C **The expression of IL-6 during hsa-miR-21-5p over-expression and CHPV infection was checked using qPCR. The expression of IL-6 was normalized to the expression of GAPDH. The expression of IL-6 got increased in hsa-miR-21-5p over-expression by 2.1 folds as compared to scramble. In hsa-miR-21-5p over-expression + CHPV, pNF-κBp65 /NF-κBp65 expression was 2.8 folds higher compared to CHPV** D **The expression of TNF-α 24 h post CHPV infection, was checked using qPCR. The expression of TNF-α was normalized to the expression of RNU6B. The expression of TNF-α got increased in hsa-miR-21-5p over-expression by 1.3 folds as compared to scramble. In hsa-miR-21-5p over-expression + CHPV, pNF-κBp65 /NF-κBp65 expression was 1.5 folds higher compared to CHPV. The experiments were performed in triplicate (n = 3) and shown as SE ± mean. One-way ANOVA has been applied using by using GraphPad-prism 8 (ver 8.3.0) to calculate the *p*-values, where: *p < 0.05, **p < 0.01, and ***p < 0.001

**Fig. 6:**
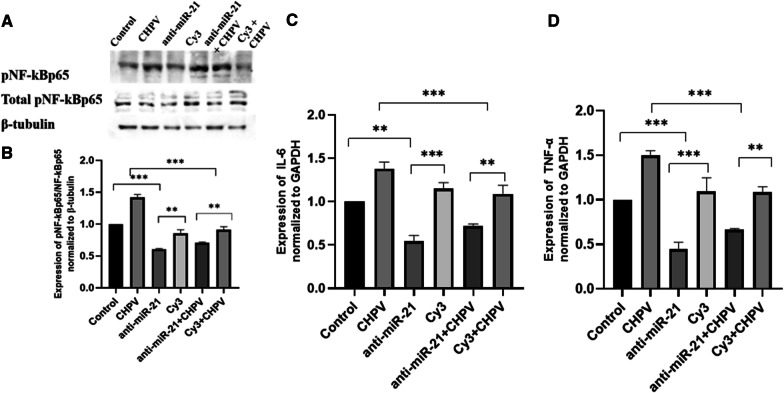
The effect of hsa-miR-21-5p knockdown on NF-κB activation. The effect of hsa-miR-21-5p on NF-κB activation was studied during hsa-miR-21-5p knockdown and CHPV infection in human microglial cells. **A **Western Blot analysis showed restoration of pNF-κB/NF-κB expression post knockdown of hsa-miR-21-5p and CHPV infection in human microglial cells. **B **The graph of densitometry analysis of pNF-κB/NF-κB showed significant suppression of pNF-κB expression by 60 % in hsa-miR-21-5p + CHPV compared to CHPV, while in hsa-miR-21-5p alone it was downregulated 25 % compared to Cy3 (**C**).The expression of IL-6 during hsa-miR-21-5p knockdown and CHPV infection was checked using qPCR. The expression of IL-6 was normalized to the expression of GAPDH. The expression of IL-6 got supressed in hsa-miR-21-5p Knockdown by 55 % as compared to Cy3. In hsa-miR-21-5p over-expression + CHPV, IL-6 expression was 60 % higher compared to CHPV (**D**). The expression of TNF-α 24 h post CHPV infection, was checked using qPCR. The expression of TNF-α was normalized to the expression of RNU6B. The expression of TNF-α got decreased in hsa-miR-21-5p knockdown by 65 % as compared to scramble. In hsa-miR-21-5p over-expression + CHPV, pNF-κBp65 /NF-κBp65 expression was 50 % supressed compared to CHPV. The experiments were performed in triplicate (n = 3) and shown as SE ± mean. One-way ANOVA has been applied using by using GraphPad-prism 8 (ver 8.3.0) to calculate the *p*-values, where: *p < 0.05, **p < 0.01, and ***p < 0.001

**Fig. 7:**
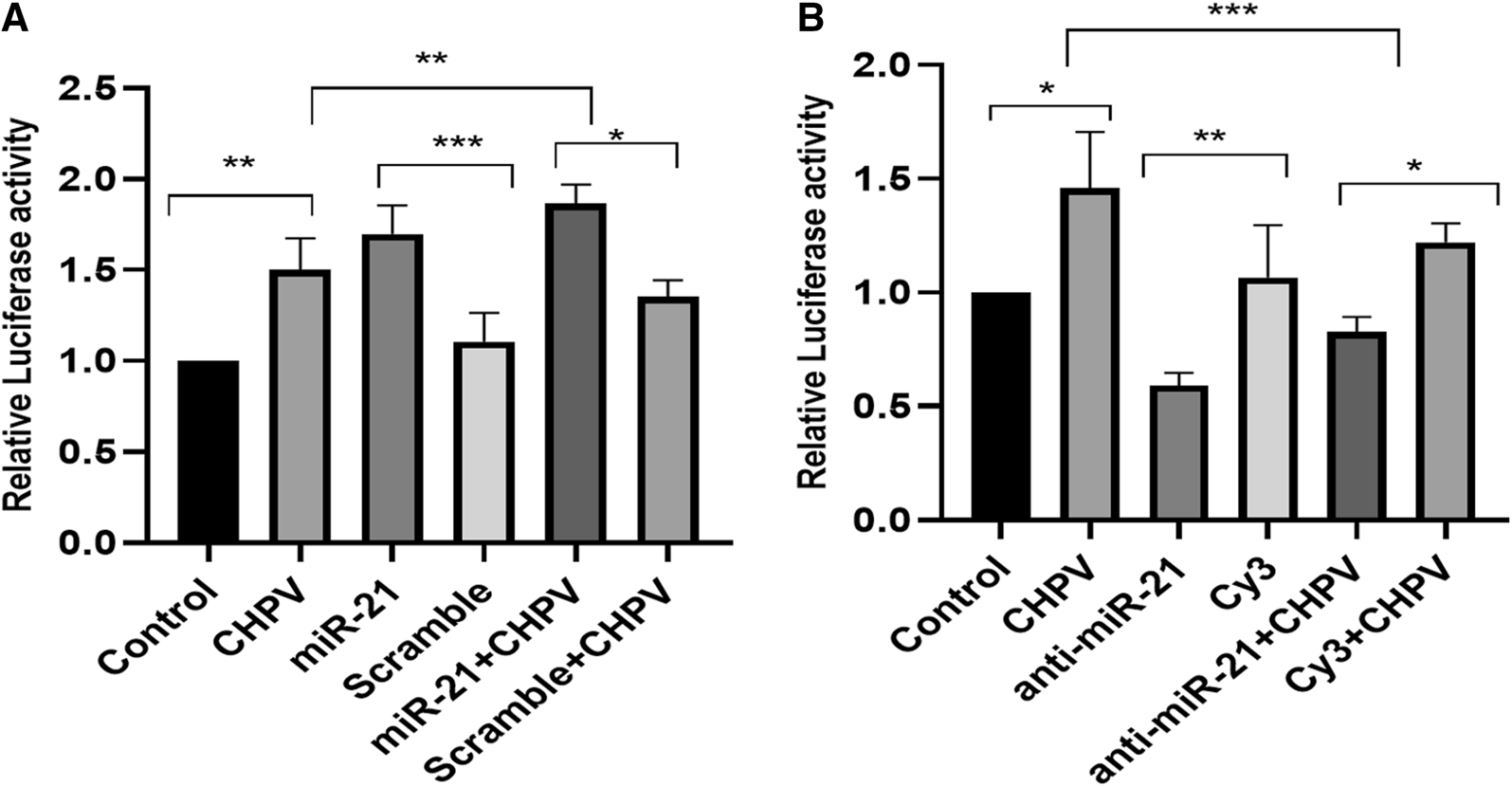
NF-κBp65 luciferase promoter activity during hsa-miR-21-5p over-expression and knockdown in human microglial cells. The human microglial cells were transfected with hsa-miR-21-5p and anti-hsa-miR-21-5p and then infected with CHPV and to see if hsa-miR-21-5p regulated the activity of NF-κB promoter.** A **The over-expression of hsa-miR-21-5p during CHPV infection increased the NF-κB-luciferase activity by 25 % compared to CHPV infected sample. The expression of NF-κB was 10 % higher in mimic alone as compared to scramble.** B **Whereas the knockdown of hsa-miR-21-5p restored the NF-κB-luciferase activity by about 25 % compared to Cy3 + CHPV. The NF-κB-luciferase activity reduced by nearly 40 % in anti-hsa-miR-21-5p as compared to Cy3

During hsa-miR-21-5p overexpression in microglial cells the expression of pNF-κBp65 /NF-κBp65 increased by ~ 60 % as compared to scramble. In hsa-miR-21-5p over-expressed + CHPV infected microglial cells, the expression of pNF-κBp65 /NF-κBp65 was ~ 45 % higher compared to CHPV infected microglial cells (Fig. 5A, B). The pNF-κBp65 /NF-κBp65 expression suppressed by ~ 25 % in anti-hsa-miR-21-5p compared to Cy3. In the presence of CHPV infection, pNF-κBp65 /NF-κBp65 expression suppressed by ~ 60 % in anti- hsa-miR-21-5p + CHPV compared to CHPV (Fig. 6A). The human microglial cells were transfected with hsa-miR-21-5p and anti-hsa-miR-21-5p and then infected with CHPV and to see if hsa-miR-21-5p regulated the activity of the NF-κB promoter. The over-expression of hsa-miR-21-5p during CHPV infection increased the NF-κB-luciferase activity by 25 % compared to CHPV infected sample (Fig. 7A). The expression of NF-κB was 10 % higher in mimic alone as compared to scramble (Fig. 7A). Whereas the knockdown of hsa-miR-21-5p in presence of CHPV restored the NF-κB-luciferase activity by about 25 % compared to CHPV infection (Fig. 7B). The NF-κB-luciferase activity reduced by nearly 40 % in anti-hsa-miR-21-5p as compared to Cy3 negative control (Fig. 7B).

The effect of hsa-miR-21-5p was studied on IL-6 and TNF-α. During hsa-miR-21-5p overexpression, the expression of IL-6 and TNF-α increased by 2.1 folds and 1.3 folds respectively as compared to scramble. In hsa-miR-21-5p over-expression + CHPV, pNF-κBp65 /NF-κBp65 expression was 2.8 folds and 1.5 folds higher respectively compared to CHPV (Fig. 5C, D). The IL-6 and TNF-α expression got suppressed by ~ 60 % and ~ 65 % respectively in anti- hsa-miR-21-5p compared to Cy3. In presence of CHPV infection, IL-6 and TNF-α expression got suppressed by ~ 55 % and ~ 50 % respectively in anti- hsa-miR-21-5p + CHPV as compared to CHPV (Fig. 6C, D).

## Discussion

Chandipura virus is a neglected RNA virus that causes acute encephalitis, mostly among children. CHPV infection leads to neuroinflammation and promotes neuronal death [3]. Microglia are the innate immune cells of the Central Nervous System (CNS) parenchyma, which play a critical role in controlling viral infections [10]. Microglial cells get activated during CHPV infection due to neuronal injury and the release of pro-inflammatory cytokines [35].

MicroRNAs are the endogenous regulators of gene expression. Many previously published studies highlighted the role of microRNAs in regulating the immune responses during viral infections [16, 18]. The increased expression of miR-155 has been reported to be associated with inflammation, microglial activation, compromise Blood Brain Barrier (BBB) permeability in various CNS disorders leading to the induction of neuroinflammation [33, 36–39]. The miR-146a on the other hand negatively regulates the inflammation and promotes viral replication [40–42]. The miR-146a also impairs inflammatory signalling in Alzheimer’s disease [43]. miR-34 has been reported to be involved in the induction of antiviral responses and decreased viral replication during JEV infection [44, 45]. It is important to understand the molecular pathogenesis of CHPV to develop therapeutics.

In this study, we demonstrated the role of hsa-miR-21-5p in regulating the inflammatory responses in CHPV infected microglial cells. CHPV infection alters the expression of hsa-miR-21-5p which in turn supresses PTEN. The suppression of PTEN by hsa-miR-21-5p activated the PI3K/AKT signalling and promoted the activation of NF-ĸB and pro-inflammatory responses during CHPV infection.

The hsa-miR-21-5p has been reported to play an important role in the regulation of immune responses [32]. Perturbation of hsa-miR-21-5p expression has been reported in viral infections [46, 47]. The increased expression of hsa-miR-21-5p has been reported in DENV2, EBV, and HCV infection in order to control the viral replication and host innate immune responses [48–50]. In this study, we observed upregulation in the expression of has-miR-21-5p upon CHPV infection in human microglial cells. To test whether the hsa-miR-21-5p expression is virus specific, we checked the expression of miR-21-5p in other neurotropic virus (JEV) as well as non-neurotropic virus (CHIKV) in human microglial cells. The JEV and CHIKV infection resulted to the increased hsa-miR-21-5p expression during late stages of infection. (Additional file 3: Fig. 3A, B). Several reports support that the miRNA expression varies in different viral infections, even the change in the virus strain affects the miRNA expression [33, 40, 51–53]. PTEN is widely known for its role as a tumour suppressor and regulation of immune responses [28, 54]. PTEN negatively regulates the PI3K/AKT signalling pathway [55]. It also inhibits the phosphorylation of Akt and enhances tumorigenesis [56]. In our study, the suppression of PTEN by hsa-miR-21-5p resulted in the increased phosphorylation of Akt as compared to controls in CHPV infected microglial cells (Fig. 1D, E). The involvement of the PI3K/AKT pathway has been reported during viral infections as an inducer of interferons and inflammation [32, 57]. PI3K/AKT pathway is also activated by viruses to slow down cell death and promote viral replication [58–61]. During JEV infection suppression of PTEN promotes phosphorylation of AKT and IRF-3 at 24 h post infection in human microglial cells [27] The role of the hsa-miR-21-5p in regulation of PTEN/PI3K/AKT pathway has also been reported in oesophageal cancer [62]. The overexpression of hsa-miR-21-5p in human microglial cells resulted in suppression of PTEN and promoted phosphorylation of AKT, whereas the use of hsa-miR-21-5p inhibitor resulted in the restoration of the PTEN and suppression of AKT phosphorylation. This observation supported the previously published report which demonstrated PTEN as a target for hsa-miR-21-5p [63]. NF-ĸB is a transcriptional factor that governs the expression of genes critical to various cellular processes including inflammation [34]. Akt phosphorylation has been reported to promote the phosphorylation of NF-ĸBp65 to induce its activation and enhance tumorigenesis [20, 34]. The activated NF-ĸBp65 translocate to the nucleus and promote the synthesis of inflammatory genes. We observed activation of NF-ĸBp65 at 24 h during CHPV infection (Fig. 2A, B). CHPV infection in mice increase the secretion of pro-inflammatory cytokines like TNF-α, MCP-1, IL-1, IL-2 and IL-6 [8, 13, 64, 65]. In our study the phosphorylation of NF-ĸBp65 enhanced expression of IL-6, TNF-α and IL-1β, 24 h post-infection (Fig. 2C–E). The over-expression of has-miR-21-5p resulted in the activation of pro-inflammatory responses in human microglial cells as it increased the phosphorylation of NF-ĸBp65 and expression of IL-6 and TNF-α (Fig. 5A–D). Inhibition of hsa-miR-21-5p resulted in the suppression of NF-ĸBp65 activation and suppression of pro-inflammatory responses (Fig. 6A–D). These results were further validated with luciferase assay to confirm the hsa-miR-21-5p mediated regulation of the NF-κB-promoter activity both during over-expression and knockdown (Fig. 7).

**Fig. 8:**
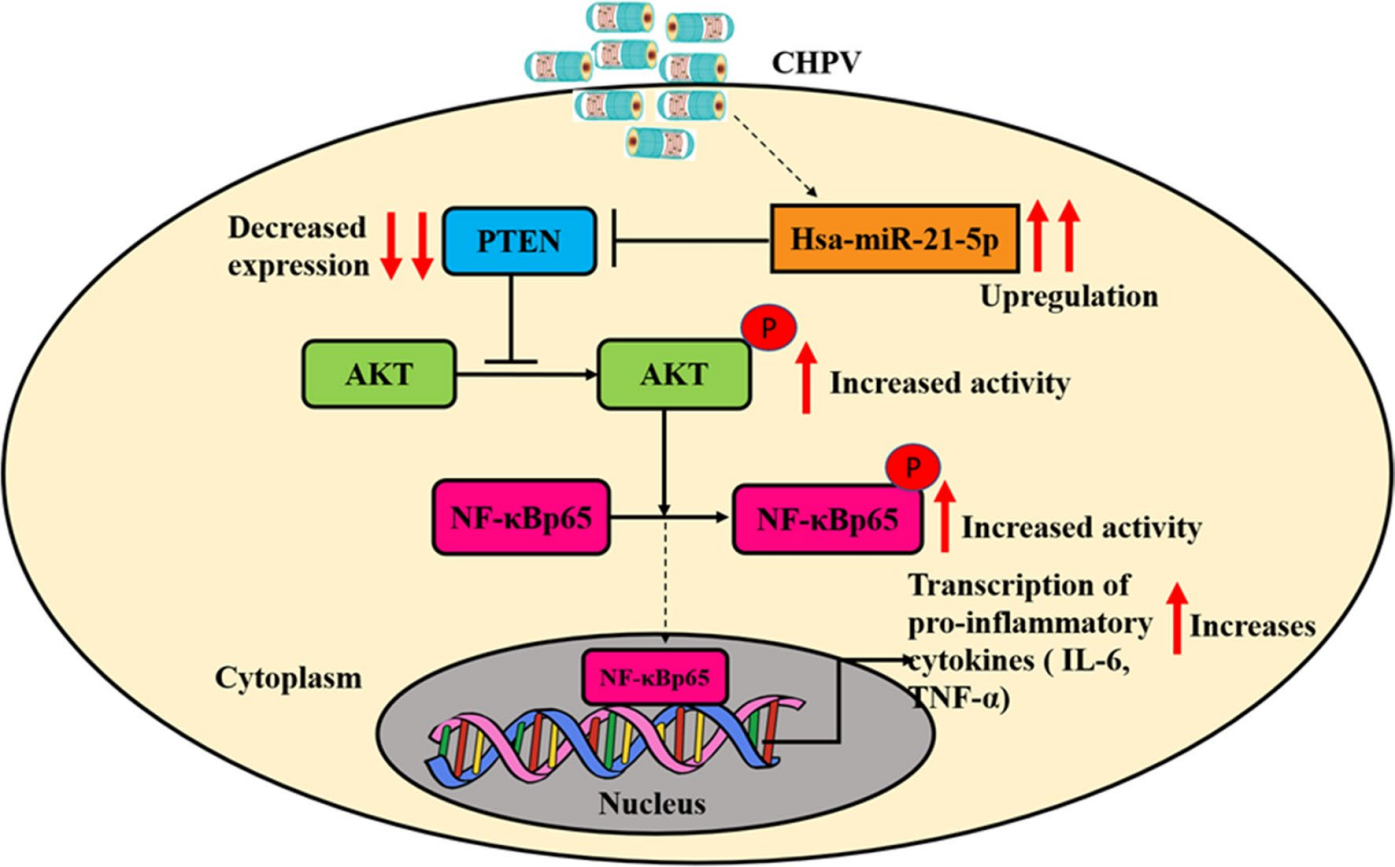
The CHPV mediated regulation of inflammatory responses by perturbation of hsa-miR-21-5p in human microglial cells. CHPV infection in human microglial cells increases the expression of hsa-miR-21-5p. The microRNA targets and supresses the expression of PTEN which in turns promotes phosphorylation and activation of AKT and NF-κBp65 in human microglial cells

In summary, the findings of this study supported, that CHPV infection led to the increased hsa-miR-21-5p expression in human microglial cells. The upregulation of miRNA suppressed the PTEN expression and promoted the phosphorylation of AKT and NF-ĸBp65 through a positive feedback loop (Fig. 8). This study highlights the crucial role of hsa-miR-21-5p during CHPV infection, which may be used in small RNA based therapeutics.

## Conclusions

The present study showed the role of hsa-miR-21-5p in activation of NF-ĸB during Chandipura virus infection in human microglial cells through PI3K/AKT pathway. The activation of NF-ĸB promotes the induction of pro-inflammatory responses and may contribute to CHPV induced neuroinflammation.

## Supplementary Information

**Additional file 1:** **Figure S1. **CHPV infection upregulates hsa-miR-21-5p which targets and reduces the expressionof PTEN. Human microglial cells were infected with CHPV at MOI 0.1 for 12, 18 and 24h. **A** The expression ofhsa-miR-21-5p was checked by qPCR at these time points. hsa- miR-21-5p was found to be upregulated significantly by ~1.6 folds at 24 hours post infection. **B** The over-expression and knockdownof hsa-miR-21-5p was confirmed by qPCR. The miR-21 expression increased more than 1000 folds in hsa-miR-21-5p mimic as compare to scramble sequence, whereas the expression of hsa-miR-21-5p was more than 90% reduced in anti-hsa-miR-21-5p as compared to Cy3. **C** Western blotting showed reduction in PTEN expression post CHPV infection in human microglial cells at 18 and 24h. **D **Densitometry analysis showed that PTEN expression decreased significantly by 40%, 24 hours post infection. **E** Western Blotting showed that PTEN expression was reduced in hsa-miR-21-5p mimic as compared to scramble sequence while it restored in anti-hsa-miR-21-5p. **F** Densitometry analysis showed that the expression of PTEN decreased by ~50% in hsa-miR-21-5p mimic as compared to scramble sequence, whereas in anti-hsa-miR-21-5p PTEN expression was ~30% higher compared to Cy3.

**Additional file 2:** **Figure S2. **CHPV infected human microglial cells. Human microglial cells were infected with CHPV at MOI 0.1. Bright Field Images were taken 12h, 18h and 24h post CHPV infection.

**Additional file 3: Figure S3. **hsa-miR-21 expression in JEV and CHIKV infected human microglial cells. Human microglial cells were infected with JEV at MOI 5 and with CHIKV at MOI 2. **A** The expression of hsa-miR-21-5p in JEV infected human microglial cells was checked by qPCR using TaqMan primers and probes specific for miR-21-5p. The expression of hsa-miR-21-5p was normalized to the expression of RNU6B and was upregulated at 24 and 48h folds by 1.5 and 1.3 folds, respectively in JEV infected human microglial cells. **B** The expression of hsa-miR-21-5p in CHIKV infected human microglial cells was checked by qPCR using TaqMan primers and probes specific for miR-21-5p. The expression of hsa-miR-21-5p was normalized to the expression of RNU6B and was downregulated by 80% at 24h, by 60% at 36h and upregulated by 2 folds at 48h during CHIKV infection in human microglial cells. The experiments were performed in triplicate (n=3) and shown as SE± mean.

## Data Availability

All data generated in this study are available from corresponding author on reasonable request.

## Abbreviations

CHPV: Chandipura virus
RNA: Ribonucleic acid
miRNA: microRNA
PTEN: Phosphatase and tensin homolog
AKT: Ak strain transforming (more commonly known as Protein kinase B (PKB))
NF-κB: Nuclear Factor Kappa light chain enhancer of activated B cells
IL-6: Interleukin 6
TNF-α: Tumour Necrosis Factor alpha
GAPDH: Glyceraldehyde 3-phosphate dehydrogenase
DMEM: Dulbecco’s Modified Eagle Medium
FBS: Fetal bovine serum
PENSTREP: Penicillin Streptomycin solution
DENV2: Dengue Virus Type 2
EBV: Epstein-Barr virus
HCV: Hepatitis C virus
JEV: Japanese encephalitis virus
CHIKV: Chikungunya virus
ANOVA: Analysis of variance
NBRC: National Brain Research Centre
