# Supplementary figures and images for: Chandipura virus dysregulates the expression of hsa-miR-21-5p to activate NF-κB in human microglial cells
Source: J Biomed Sci. 2021 Jul 7;28:52. doi: 10.1186/s12929-021-00748-0 (PMC8265105; doi:10.1186/s12929-021-00748-0)

## Slide 1
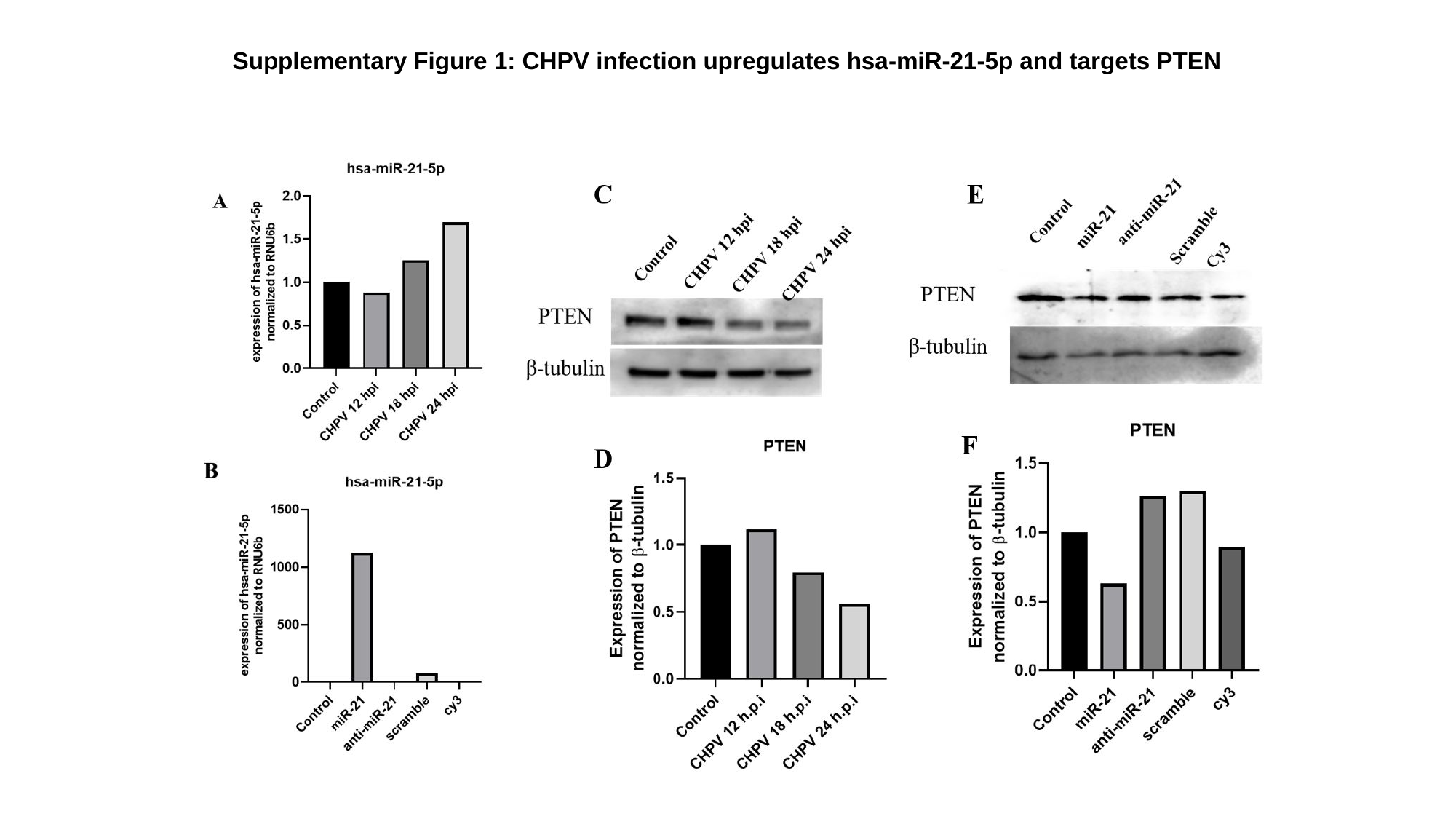

Supplementary Figure 1: CHPV infection upregulates hsa-miR-21-5p and targets PTEN

Supplement: Supplementary file 1 — Additional file 1: Figure S1. CHPV infection upregulates hsa-miR-21-5p which targets and reduces the expressionof PTEN. Human microglial cells were infected with CHPV at MOI 0.1 for 12, 18 and 24h. A The expression ofhsa-miR-21-5p was checked by qPCR at these time points. hsa- miR-21-5p was found to be upregulated significantly by ~1.6 folds at 24 hours post infection. B The over-expression and knockdownof hsa-miR-21-5p was confirmed by qPCR. The miR-21 expression increased more than 1000 folds in hsa-miR-21-5p mimic as compare to scramble sequence, whereas the expression of hsa-miR-21-5p was more than 90% reduced in anti-hsa-miR-21-5p as compared to Cy3. C Western blotting showed reduction in PTEN expression post CHPV infection in human microglial cells at 18 and 24h. D Densitometry analysis showed that PTEN expression decreased significantly by 40%, 24 hours post infection. E Western Blotting showed that PTEN expression was reduced in hsa-miR-21-5p mimic as compared to scramble sequence while it restored in anti-hsa-miR-21-5p. F Densitometry analysis showed that the expression of PTEN decreased by ~50% in hsa-miR-21-5p mimic as compared to scramble sequence, whereas in anti-hsa-miR-21-5p PTEN expression was ~30% higher compared to Cy3. [file 12929_2021_748_MOESM1_ESM.pptx]

## Slide 1
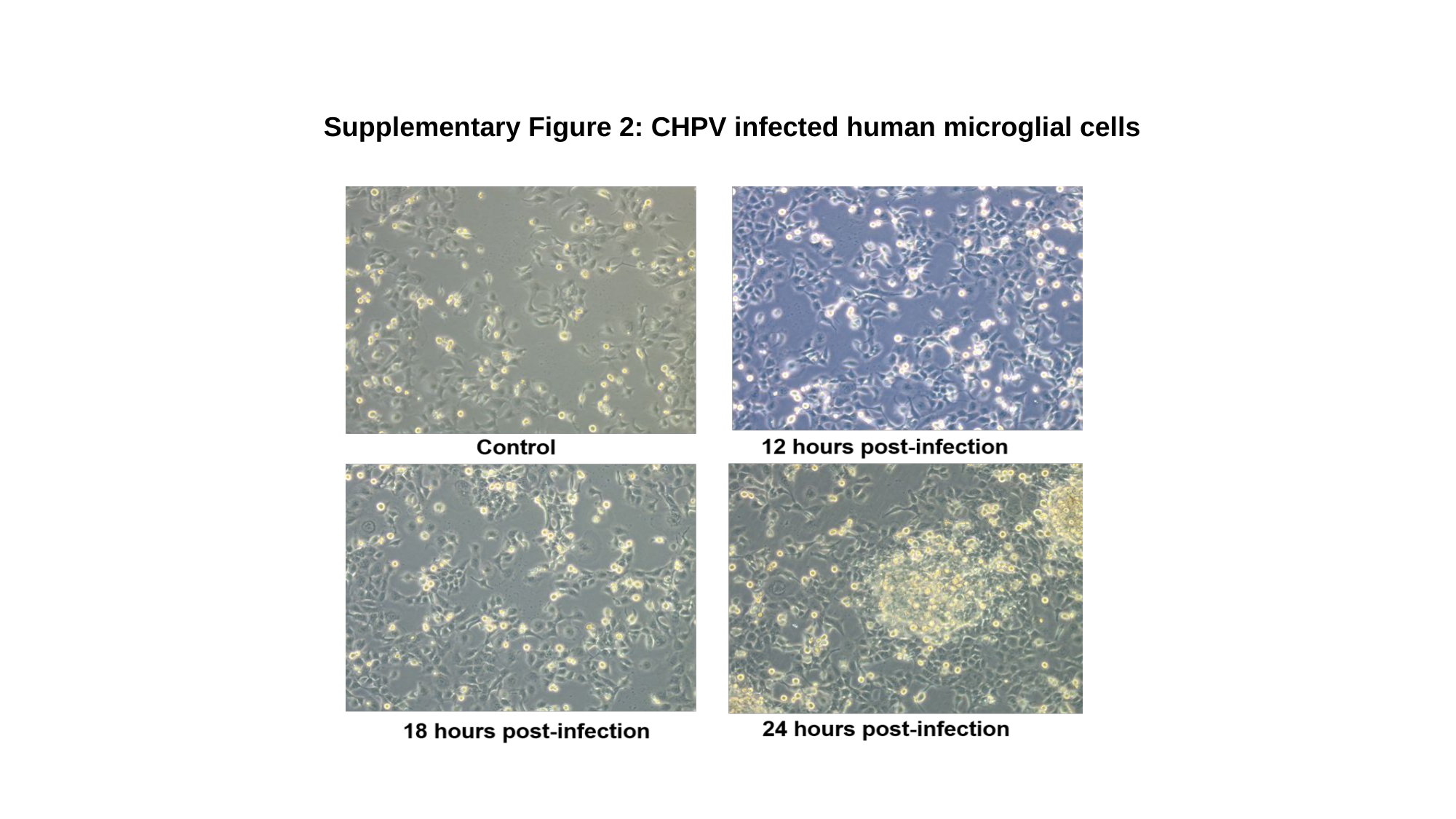

Supplementary Figure 2: CHPV infected human microglial cells

Supplement: Supplementary file 2 — Additional file 2: Figure S2. CHPV infected human microglial cells. Human microglial cells were infected with CHPV at MOI 0.1. Bright Field Images were taken 12h, 18h and 24h post CHPV infection. [file 12929_2021_748_MOESM2_ESM.pptx]
