# Supplementary material for: Chandipura virus dysregulates the expression of hsa-miR-21-5p to activate NF-κB in human microglial cells
Source: J Biomed Sci. 2021 Jul 7;28:52. doi: 10.1186/s12929-021-00748-0 (PMC8265105; doi:10.1186/s12929-021-00748-0)
Supplement: Supplementary file 3 — Additional file 3: Figure S3. hsa-miR-21 expression in JEV and CHIKV infected human microglial cells. Human microglial cells were infected with JEV at MOI 5 and with CHIKV at MOI 2. A The expression of hsa-miR-21-5p in JEV infected human microglial cells was checked by qPCR using TaqMan primers and probes specific for miR-21-5p. The expression of hsa-miR-21-5p was normalized to the expression of RNU6B and was upregulated at 24 and 48h folds by 1.5 and 1.3 folds, respectively in JEV infected human microglial cells. B The expression of hsa-miR-21-5p in CHIKV infected human microglial cells was checked by qPCR using TaqMan primers and probes specific for miR-21-5p. The expression of hsa-miR-21-5p was normalized to the expression of RNU6B and was downregulated by 80% at 24h, by 60% at 36h and upregulated by 2 folds at 48h during CHIKV infection in human microglial cells. The experiments were performed in triplicate (n=3) and shown as SE± mean. [file 12929_2021_748_MOESM3_ESM.pptx]

## Slide 1
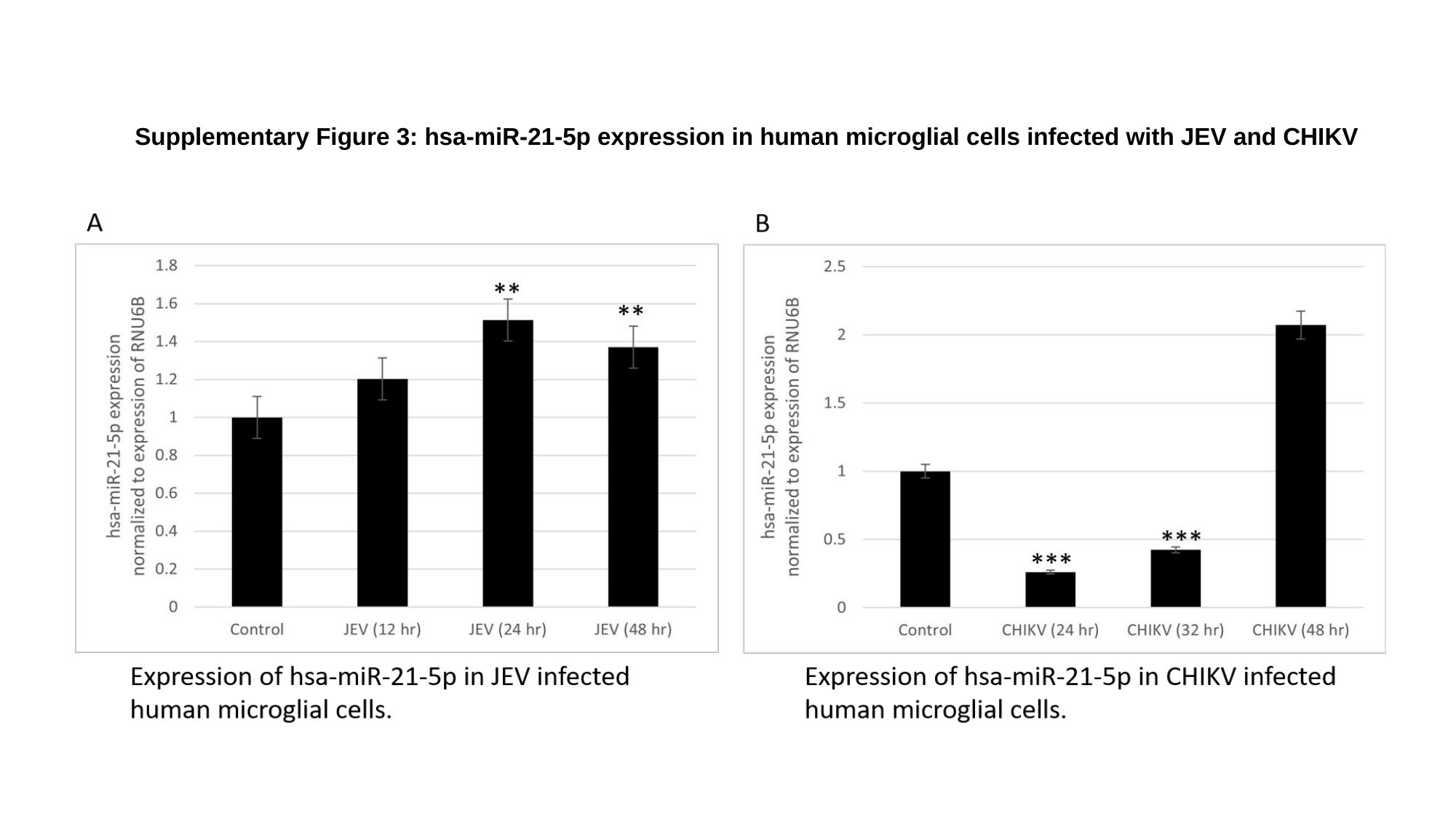

Supplementary Figure 3: hsa-miR-21-5p expression in human microglial cells infected with JEV and CHIKV
